# Stellate Ganglion Block in the Treatment of Long COVID: A Systematic Review

**DOI:** 10.1007/s11916-026-01477-5

**Published:** 2026-04-13

**Authors:** Sudeep Peddireddy, Noah VanWingerden, Priya Patel, Gabriel Howard, Jeffrey Berger

**Affiliations:** 1 Temple University, 3401 N. Broad St, Philadelphia, PA 19140 USA; 2 Premier Orthopedics, 1204 Baltimore Pike , Chadds Ford, PA 19317 USA

**Keywords:** Post Acute Sequelae of SARS-CoV-2, Long COVID, Stellate ganglion block, Autonomic dysfunction, Treatment efficacy, Systematic review

## Abstract

**Objectives:**

This review evaluates stellate ganglion block as a treatment for long COVID, seeking to evaluate the treatment’s efficacy by various symptoms and the limitations of the current literature.

**Study Design:**

Systematic Review.

**Setting:**

Ambulatory or Outpatient Setting.

**Methods, Subjects:**

A systematic review of the current literature regarding use of stellate ganglion block in patients with long COVID was conducted. 2 databases were searched on August 28th, 2025. Search terms were “long COVID” and “stellate ganglion block”, yielding 45 results. Studies examining patient outcomes after stellate ganglion block were included. Case reports, case series, basic science studies and previous reviews were excluded. Seven studies met inclusion criteria.

**Results:**

Patients received a single stellate ganglion block in some studies and multiple stellate ganglion blocks in others. All studies reported symptomatic improvement without control groups. Response rates ranged from 55.8% to 100%. The most robust improvements (> 80% patients reporting relief) were seen in cough, dyspnea, headache, joint pain, pain interference/intensity, pins/needles, subjective relief.

**Conclusion:**

Stellate ganglion block is a promising treatment that appears to generate substantive benefit for many of the symptoms seen in long COVID. However, the current literature has small, uncontrolled studies with heterogenous study designs and follow-up periods. Standardized research with larger sample sizes, control groups, and longer-term follow up is necessary to elucidate the degree of benefit.

IRB approval and clinical trial registration not required.

## Introduction

The global COVID-19 pandemic introduced enormous strain into healthcare systems worldwide which remains challenging to qualify and quantify even several years after the initial outbreak. Patients and healthcare workers alike have reported degradations in their quality of life which has been directly and indirectly linked with the COVID-19 pandemic [1, 2]. Although most COVID-19 symptoms are often experienced over a number of days to weeks, the development of post-acute sequelae of COVID-19 or “*long COVID”* has been described by various authors since 2020 [3]. The World Health Organization (WHO) currently describes long COVID as the “continuation or development of new symptoms 3 months after the initial SARS-CoV-2 infection”, with “over 200 different symptoms have been reported that can have an impact on everyday functioning [4].”

The mechanism of long COVID is not fully understood, however, many prominent theories suggest that COVID-19 induces a systemic inflammatory reaction which results in autonomic nervous system dysregulation, glial cell activation, blood brain barrier degradation and activation of other damaging neurochemical cascades [5]. Due to a significant degree of neurologic dysregulation, blockade of the autonomic nervous system has been utilized to “reset” maladaptive neurologic pathways and reduce patient symptom burden. The stellate ganglion is a cervical autonomic ganglion and stellate ganglion blocks (SGB) have been reported to provide symptomatic relief for those with long COVID [6].

Mainstays of treatment for long COVID are symptom based and patient focused. The American Academy of Physical Medicine and Rehabilitation (AAPM&R) encourages treatment of long COVID in a holistic, patient-centered manner through the use of detailed physician assessment, therapy, as well as pharmacologic management as indicated for symptomatic treatment.^6^ In addition to medical society guidelines, other initiatives such as *RECOVER*, seek to compile patient cohort data and provide clinical trials to strengthen literature, which are ongoing [7]. SGBs have been used for other dysregulatory syndromes such as chronic regional pain syndrome (CRPS), post traumatic stress disorder (PTSD), and ventricular tachycardia [8]. Although poorly understood, SGBs have been postulated to reset a maladaptive neurologic feedback loop in each of these medical conditions [9]. Disruptions of such negative feedback loops, as also seen in long COVID, with SGBs have reportedly provided symptomatic relief.

Despite significant effort, gaps remain in the medical literature regarding the efficacy and availability of interventions such as SGB for patients with long COVID. Furthermore, aggregation of data regarding particular symptom relief after SGB within the long COVID symptom milieu remains scarce. The goal of this review is to critically evaluate current data for the use of SGBs for patients with long COVID, to assess symptom-specific efficacy, identify current research limitations and identify further study needs.

## Methods

Two databases, EMBase and PubMed, were queried with the search terms “stellate ganglion block” and “long COVID”. This search was performed on August 28th, 2025 for all-time publication data without initial screening for geography, date, or primary language. This search yielded a total of 45 results between both databases. Articles were subsequently reviewed under strict inclusion/exclusion criteria by title name and abstract, then by full-text review if deemed appropriate after title/abstract review. To be included, studies must examine outcomes of patients diagnosed with long COVID after at least one SGB, be written in English, and have the full-text article available. Of the 45 results, the following were excluded: 14 duplicate results, 12 case reports/series, 4 unpublished/ongoing clinical trials, 2 articles examining unrelated therapies (ketamine, OMT), and 6 reviews/clinical guidelines that were not specific to SGB in the setting of long COVID. A total of seven studies met the inclusion criteria. Four reviewers examined each article, noting patient population, intervention, key dependent variables, outcome, and limitations of each study. Findings were organized by symptom.


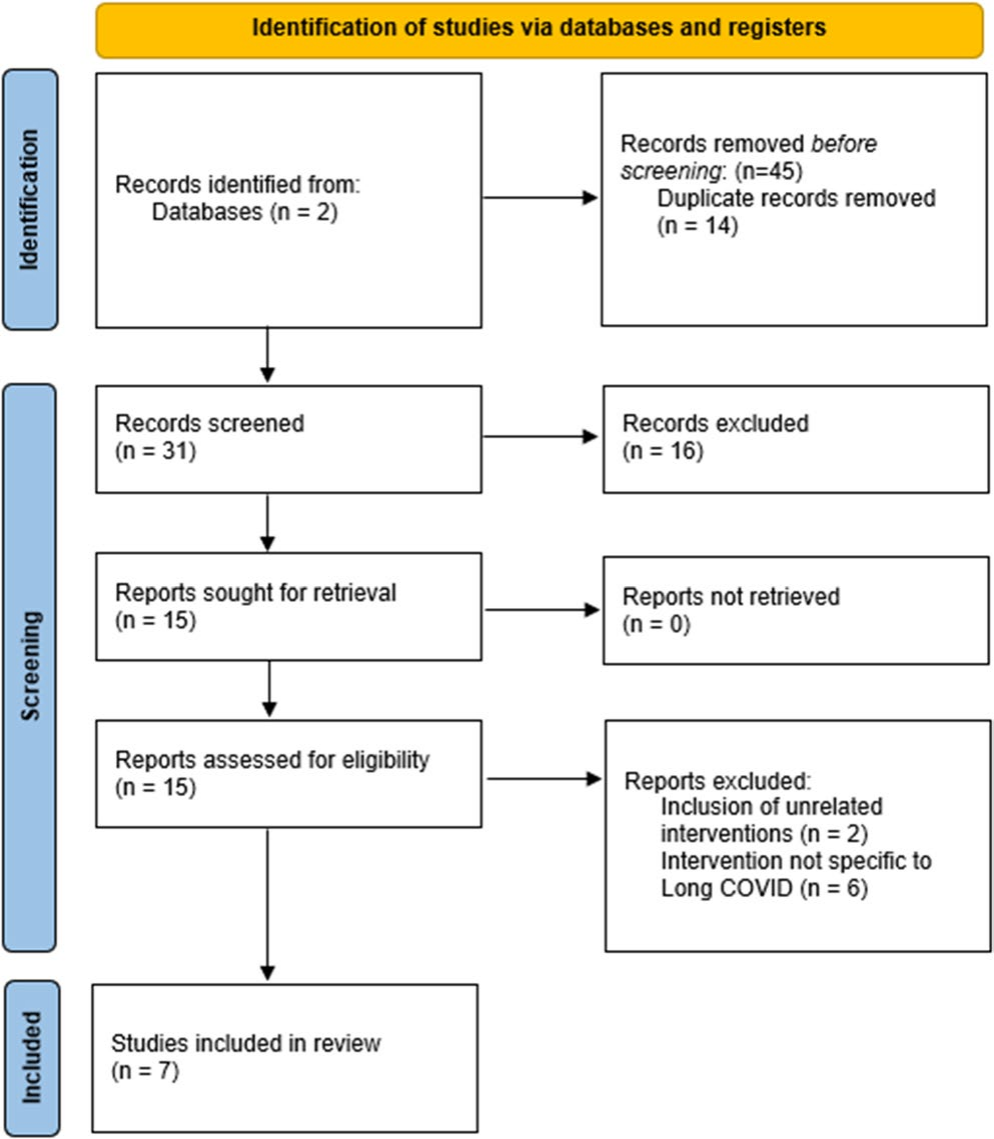


## Results

### Study Characteristics

Seven studies met the inclusion criteria. Protocols varied widely between the studies, with some offering unilateral SGB while others offered multiple bilateral SGB over several weeks. There was also a disparity with each study’s follow up period. No study had a control group for comparison. Most symptoms and subsequent improvements were self-reported with some exceptions as outlined below (Table 1). Symptoms were also organized by number of papers that studied that symptom and gross efficacy after SGB intervention represented by a pooled percentage of patients that reported benefit (Table 2).

**Table 1:** Various symptoms assessed after stellate ganglion blocks with relevant papers, their corresponding treatments and their reported outcomes

Symptom	Papers	Intervention	Major Findings
Anosmia/Parosmia/Ageusia	[14] [17] [10] [13]	Either right-sided SGB or bilateral (right, then left) SGB Unspecified number of SGB Bilateral SGB within 48 h Unilateral SGB with a contralateral SGB offered 1–2 weeks later	10/18 patients reported resolution of changes in taste/smell 15/33 patients reported improvement in parosmia; 7/15 patients reported sustained improvement of parosmia 7/9 patients with improved loss of smell. 6/8 patients reported improvement in loss of smell
Anxiety	[15] [16] [10] [13]	Single SGB Right side SGB on initial visit, then left side SGB 1 week later Bilateral SGB Unilateral SGB with a contralateral SGB offered 1–2 weeks later	No significant change in GAD-7 score 9/16 patients reported improved anxiety 6/6 patients reported improvement in anxiety 7/10 patient reported improved anxiety
Autonomic Dysfunction	[15] [16] [14] [11]	Single SGB Right side SGB on initial visit, then left side SGB 1 week later Either right-sided SGB or bilateral (right, then left) SGB Bilateral SGB weekly for 3 weeks (6 SGB total)	10 beats per minute decrease in heart rate (significantly improved) 13/17 patients showed improvement of autonomic dysfunction via COMPASS-31 scores 7/9 patients reported resolution of tachycardia/palpitations 3/4 patients had resolution of orthostatic hypotension
Brain Fog/Cognitive Dysfunction	[14] {13] [11]	Either right-sided SGB or bilateral (right, then left) SGB Unilateral SGB with a contralateral SGB offered 1–2 weeks later Bilateral SGB weekly for 3 weeks (6 SGB total)	26/33 patients reported resolution of brain fog 25/38 of patients reported improvement in brain fog 7/10 patients showed improvements in combined *BrainCheck* cognitive domain testing
Chest Pain	[14] [13]	Either right-sided SGB or bilateral (right, then left) SGB Unilateral SGB with a contralateral SGB offered 1–2 weeks later	8/10 patients reported resolution of chest pain 4/10 patients reported improvement in chest pain
Cortisol Level	[11]	Bilateral SGB weekly for 3 weeks (6 SGB total)	No change in morning cortisol
Cough	[14]	Either right-sided SGB or bilateral (right, then left) SGB	8/10 patients reported resolution of cough
Depression/Mood changes	[16] [14] [10] [11]	Right side SGB on initial visit, then left side SGB 1 week later Either right-sided SGB or bilateral (right, then left) SGB Bilateral SGB’s Bilateral SGB weekly for 3 weeks (6 SGB total)	6/16 patients reported improved depression 16/21 patients reported resolution of mood changes 5/5 patients reported improvement in depression 9/10 showed improved “Vitality” on SF-36 questionnaire
Diarrhea	[14] [13]	Either right-sided SGB or bilateral (right, then left) SGB Unilateral SGB with a contralateral SGB offered 1–2 weeks later	7/8 patients reported resolution of diarrhea 2/6 patients reported improvement in GI symptoms
Dizziness	[14] [10] [13]	Either right-sided SGB or bilateral (right, then left) SGB Bilateral SGB’s Unilateral SGB with a contralateral SGB offered 1–2 weeks later	13/17 patients reported resolution of dizziness 5/5 patients reported improvement in dizziness upon standing 10/18 patients reports improvement in general dizziness
Dyspnea	[14]	Either right-sided SGB or bilateral (right, then left) SGB	15/17 patients reported resolution of dyspnea
Fatigue	[15] [16] [14] [10] [13] [11]	Single SGB Right side SGB on initial visit, then left side SGB 1 week later Either right-sided SGB or bilateral (right, then left) SGB Bilateral SGB’s Unilateral SGB with a contralateral SGB offered 1–2 weeks later Bilateral SGB weekly for 3 weeks (6 SGB total)	9-point decrease in median FSS (significantly improved) 8/16 patients had improved fatigue via PROMIS-29 score at 4 weeks 27/35 patients reported resolution of fatigue 7/7 patients reported improvement in fatigue 22/38 patients reported improvement in fatigue 10/10 showed improvement in physical function and social function on SF-36 questionnaire
Headache	[14] [13]	Either right-sided SGB or bilateral (right, then left) SGB Unilateral SGB with a contralateral SGB offered 1–2 weeks later	13/16 patients reported resolution of headache 13/16 patients reported improvement in headache
Joint Pain	[14]	Either right-sided SGB or bilateral (right, then left) SGB	15/16 patients reported resolution of joint pain
Miscellaneous (Rash, fever, menstrual cycle changes)	[14]	Either right-sided SGB or bilateral (right, then left) SGB	1/1 patient reported resolution of fever 2/2 patients reported resolution of rash 4/5 patients reported resolution of menstrual cycle changes
Pain Interference/Intensity	[16]	Right side SGB on initial visit, then left side SGB 1 week later	13/16 patients reported improvement of pain interference and pain intensity via PROMIS-29 scores at 4 weeks
Post-exertional malaise	[11] [13] [11]	Bilateral SGB Unilateral SGB with a contralateral SGB offered 1–2 weeks later Bilateral SGB weekly for 3 weeks (6 SGB total)	7/7 patients reported improvement in post exertional malaise 11/25 patients reported improvement in post exertional malaise Statistically significant cohort improvement in post-exertional malaise
Pins/Needles	[14]	Either Right-sided SGB or bilateral (right, then left) SGB	9/9 patients reported resolution of pins/needles
Post Traumatic Stress Disorder	[15	Single SGB	No significant change in PCL-5 score
Sleep	[16] [14] [11] [13] [11]	Right side SGB on initial visit, then left side SGB 1 week later Either Right-sided SGB or bilateral (right, then left) SGB Bilateral SGB’s Unilateral SGB with a contralateral SGB offered 1–2 weeks later Bilateral SGB weekly for 3 weeks (6 SGB total)	16/17 patients reported subjective improvement in sleep; PROMIS-29 sleep scores significantly improved at 1 and 4 weeks 10/14 patients reported resolution of difficulty sleeping 7/7 patient reported improvement in sleep problems 6/16 reported improvement in sleep disturbance No improvement in sleep assessments of wakefulness after sleep onset, total sleep time, percent REM sleep, sleep fragmentation, and sleep apnea using SleepImage System ring device
Subjective Relief	[15] [16]	Single SGB Right side SGB on initial visit, then left side SGB 1 week later	7/8 patients seen at followup reported subjective improvement for at least 6 weeks 15/17 patients reported subjective symptom improvement at 4 weeks

**Table 2:** Condensed table of all assessed symptoms, with count of relevant papers and gross efficacy, reported as a pooled percentage of patients that reported benefit after the intervention. The symbol * designates a “highly responsive” symptom, which is defined as a symptom which is reported in at least three of the included studies with at least 60% of all study participants reporting improvement in this domain

Symptom	Papers	Major Findings
Anxiety*	4 of 7 papers	22/32 = 68.8% of patient reported improvement in anxiety Note: 1 paper noted no significant change in GAD-7 score
Autonomic Dysfunction*	4 of 7 papers	23/30 = 76.7% of patients reported improvements in autonomic dysfunction symptoms Note: 1 study showed a statistically significant decrease in heart rate by 10 beat per minute
Brain Fog/Cognitive Dysfunction*	3 of 7 papers	58/81 = 71.6% of patients reported improvement in brain fog symptoms Note: 1 study used *BrainCheck* cognitive domain testing and 7/10 patients demonstrated improvement
Chest Pain	2 of 7 papers	12/20 = 60% of patients reported improvement in chest pain
Cortisol Level	1 of 7 papers	Note: No change in morning cortisol levels found
Cough	1 of 7 papers	8/10 = 80% of patients reported resolution of cough
Depression/Mood changes*	4 of 7 papers	36/52 = 69.2% of patients reported improvement in depression/mood symptoms Note: 1 study used the SF-36 questionnaire and 9/10 patients demonstrated improvement.
Dizziness*	3 of 7 papers	28/40 = 70% of patients reported improvement of dizziness
Dyspnea	1 of 7 papers	15/17 = 88.2% of patients reported improvement in dyspnea
Fatigue*	6 of 7 papers	74/106 = 69.8% of patients reported improvement in fatigue Note: 1 study showed a significant decrease of 9 points in median Fatigue Severity Scale (FSS) scores. 2 studies used validated measures including PROMIS-29 and SF-36.
GI Changes	2 of 7 papers	9/14 = 64.3% of patients reported improvement in GI symptoms
Headache	2 of 7 papers	26/32 = 81.3% of patients reported improvement in headache
Joint Pain	1 of 7 papers	15/16 = 93.8% patients reported resolution of joint pain
Miscellaneous Symptoms	1 of 7 papers	1/1 = 100% of patients reported resolution of fever 2/2 = 100% of patients reported resolution of rash 4/5 = 80% of patients reported resolution of menstrual cycle changes
Pain Interference/Intensity	1 of 7 papers	13/16 = 81.3% patients reported improvement of pain interference and pain intensity via PROMIS-29 scores
Pins/Needles	1 of 7 papers	9/9 = 100% of patients reported resolution of numbness/tingling
Post-Exertional Malaise	3 of 7 papers	18/32 = 56.3% of patients reported resolution of post-exertional malaise.
Post Traumatic Stress Disorder	1 of 7 papers	Note: No significant change between pre- and post-intervention PCL-5 score.
Sleep*	5 of 7 papers	39/54 = 72.2% of patients reported an improvement in sleep-related problems. Note: 1 study did not find significant improvements in sleep assessments of wakefulness after sleep onset, total sleep time, percent REM sleep, sleep fragmentation, and sleep apnea using SleepImage System ring device. 1 study found significant improvements of PROMIS-29 sleep scores at 1 and 4 weeks
Smell/Taste Changes	4 of 7 papers	38/68 = 55.8% of patients reported improvement in changes in taste/smell
Subjective Relief	2 of 7 papers	22/25 = 88% of patients reported subjective relief of symptoms

### Symptoms

#### Symptom Cluster 1: Cognitive and Psychiatric

Brain fog, depression/mood changes, anxiety, and PTSD were evaluated by 3, 4, 4, and 1 studies showing 71.6%, 69.2%, 68.6% improvement, and no significant improvement, respectively. Tools such as BrainCheck, SF-36, GAD-7, PCL-5 were utilized to determine improvement in selected manuscripts; otherwise improvements were all self-reported.

#### Symptom Cluster 2: Pain

Chest pain, headache, pain interference/intensity, joint pain, pins and needles were evaluated by 2, 2, 1, 1, and 1 studies showing 60%, 81.3%, 81.3%, 93.8%, and 100% improvement respectively. Tools such as PROMIS-29 pain subscores were utilized to determine improvement in selected manuscripts; otherwise improvements were all self-reported.

#### Symptom Cluster 3: Energy

Fatigue, post-exertional malaise, and sleep were evaluated by 6, 3, and 5 studies showing 69.8%, 56.3%, 72.2% improvement respectively. Tools such as SF-36, PROMIS-29 sleep subscores, and SleepImage System ring sleep assessments of wakefulness after sleep onset, total sleep time, percent REM sleep, sleep fragmentation, and sleep apnea were utilized to determine improvement in selected manuscripts; otherwise improvements were all self-reported.

#### Symptom Cluster 4: Neurological

Changes in smell/taste, autonomic dysfunction, and dizziness were evaluated by 4, 4, and 3 studies showing 55.8%, 76.7%, and 70% improvement respectively. Tools such as the COMPASS-31 and heart rate measurements were utilized to determine improvement in selected manuscripts; otherwise improvements were all self-reported.

#### Symptom Cluster 5: Respiratory

Cough and dyspnea were evaluated by 1 study showing 80% and 88.2% improvement, respectively. All improvements were self-reported.

#### Symptom Cluster 6: Miscellaneous

Cortisol level, GI symptoms, subjective relief, rash, fever, and menstrual cycle changes were evaluated by 1, 2, 2, 1, 1, 1 studies showing no significant change, 64.3%, 88%, 100%, 100%, 80% improvement, respectively. Tools such as morning salivary cortisol were utilized to determine improvement in selected manuscripts; otherwise improvements were all self-reported.

## Discussion

### Pathophysiology

The exact pathological mechanism by which the SARS-CoV-2 virus manifests as long COVID syndrome is poorly understood. However, the virus is thought to exert its effects primarily through spike and nucleocapsid proteins that can bind most tissue types and initiate inflammatory as well as immunological cascades [12]. The symptoms produced by viral activation of such cascades will be discussed at length, particularly regarding symptom response to SGB. There is asymmetry in SGB effect for varying COVID symptoms which likely represents the ability of SGB to effectively modulate some systems while leaving other systems either indirectly affected or completely unaffected. The mechanism of SGBs and subsequent cervical autonomic blockade does seem to correspond with intuitive relief of neurologic symptoms in some individuals, such as brain fog, mood, orthostasis, and headaches. Relief of other symptoms, such as joint pain, gastrointestinal dysregulation, and others is less intuitive despite similar levels of reported benefit (93% and 64% respectively). We have identified seven symptoms of long COVID which are highly responsive to SGBs including anxiety, depression, autonomic dysfunction, brain fog, dizziness, fatigue, and poor sleep. In order to be designated as “highly responsive”, symptom response must have been reported in at least three of the included studies with at least 60% of all study participants reporting improvement in this domain (Table 2). This pooled percentage method is done as an attempt to provide a clinically meaningful benchmark for physicians to use as a means for assessing patient appropriateness for the SGB intervention; formal meta-analysis was deferred given the wide discrepancy in study design. Each of the highly responsive symptoms bears resemblance to other conditions which are known to respond to SGBs, such as PTSD, CRPS, and cardiac arrhythmias [9]. Blockade of the cervical autonomic chain is thought to disrupt maladaptive sympathetic nerve pathways in these conditions and may be similarly responsible for relief in the identified highly responsive symptoms. Anxiety, depression, brain fog and fatigue symptoms are reminiscent of the SGB indication of PTSD while poor sleep and dizziness may be more similar to autonomic dysfunction indications. Additionally, some lower responding symptoms such as chest pain or cortisol levels, may not have any clear link. Nevertheless, any comparison between highly responsive symptoms and current SGB indications is merely speculative and will be discussed further in each subheading.

### Symptom Cluster 1: Cognitive and Psychiatric

#### Brain Fog/Cognitive Dysfunction

Three studies examined cognitive dysfunction, including brain fog, from long COVID after SGB, but the treatment protocols varied substantially [11, 13, 14]. Pearson et al. performed either a unilateral right-sided SGB or bilateral (right followed by left) SGBs, whereas Chiang et al. performed a unilateral SGB with a contralateral block offered 1–2 weeks later. There was no preferential starting side for the block in Chiang et al.’s study and the rationale for side selection or criteria for receiving the contralateral block were not specified. Meanwhile, Duricka et al. [11] utilized a more standardized approach by performing bilateral SGBs weekly for 3 consecutive weeks, for a total of six SGBs. Although Duricka et al. [11] incorporated BrainCheck cognitive testing, ultimately, most outcomes were based on patients’ self-reported improvement via surveys/questionnaires, introducing potential reporting bias. Despite these methodological differences, there was an overall 71.6% improvement in brain fog/cognitive dysfunction after SGB between all of the studies.

#### Depression/Anxiety/PTSD

Four studies investigated mood changes, including depression, from long COVID after SGB [10, 11, 15, 16]. Most patients across studies received bilateral SGBs in some capacity, though Pearson et al. did include some unilateral right-sided cases. Pearson et al. and Wang et al. both initiated treatment on the right side, suggesting that laterality may influence therapeutic efficacy. Outcomes were obtained via self-reported surveys/questionnaires, but despite this limitation, there was still an overall 69.2% improvement in mood and depression after SGB. However, Levey et al. did not find any improvement in PTSD after unilateral SGB [15]. Similarly, studies demonstrated that SGB effects on anxiety had a mixed positive trend overall [10, 13, 15, 16]. Only Levey et al. showed no benefit, though notably it was the only study to formally assess anxiety with GAD-7 scoring while others subjectively reported improvement. Notably, Wang et al. showed significant benefit but it did exclude severe anxiety as a part of the exclusionary criteria, which may skew results. This may suggest improvement of anxiety generally following SGB but warrants a standardized approach with more robust, formalized testing such as serial validated anxiety measurement tools.

## Symptom Cluster 2: Pain

### Chest Pain

Two studies examined chest pain related to long COVID [13, 14]. Pearson et al. used a single SGB or bilateral SGB while Chiang et al. used a unilateral SGB with a contralateral SGB offered 1–2 weeks later. Interestingly, 8 of 10 patients reported resolution of chest pain after the single SGB intervention while only 4 of 10 patients reported improvement in chest pain with the offering of multiple SGBs. It is unclear if the patients with chest pain in Chiang et al.’s study utilized the offered contralateral SGB after the initial intervention, which may explain the variance in efficacy between the two studies. Between both studies, 60% of the patients noted improvement of chest pain.

### Headache

Two studies examined headaches from long COVID [13, 14]. Pearson et al. used a single SGB or bilateral SGB, and 13 of 16 patients reported resolution of headache. Chiang et al. used a unilateral SGB with a contralateral SGB offered 1–2 weeks later, and 13 of 16 patients also reported improvement of headache. Again, it is unclear how many patients with headache, if any, received multiple SGBs in Chiang et al.’s study. However, it is interesting that the same percentage of patients reported a benefit in both studies. Between both studies, 81% of the patients reported improvement in headache.

### Pain Interference/Intensity

Pain interference and intensity were among the least evaluated symptoms of long COVID after SGB. Only one study in this review assessed pain outcomes, using PROMIS-29 scores collected four weeks post-procedure, after a right-sided SGB followed by a left-sided block one week later [16]. The study lacked a control group and outcomes were subjective patient reports. Regardless, there was an overwhelming 81.3% improvement in pain after the interventions.

### Joint Pain

Pearson et al. evaluated joint pain improvement, with 15 of 16 (93.8%) patients reporting improvement of symptoms after SGB, but the protocol regarding which patients received unilateral or bilateral injections was not specified [14]. It is unclear whether the joint pain was diffuse or localized to certain joints as well, and if there was concomitant overlap with pre-existing arthralgic complaints (i.e. osteoarthritis, rheumatoid arthritis, hypermobility syndromes, etc.)

### Pins and Needles

Pearson et al. evaluated the effect of SGBs on “pins and needle” sensations, with 100% patients reporting resolution of symptoms [14]. Again, this study did not specify whether unilateral or bilateral injections were given to these patients. It also did not delineate if these dysesthesias were separate from pre-existing complaints such as peripheral neuropathy.

## Symptom Cluster 3: Energy

### Fatigue

Fatigue was one of the most frequently evaluated symptoms of long COVID treated with SGB, assessed across six studies in this review [10, 11, 13, 15, 16]. Protocols varied considerably as some studies performed a single SGB, while others used either sequential or bilateral blocks. Laterality preference was inconsistently defined: Levey et al. and Chiang et al. did not specify a preferred side, while Wang et al. and Pearson et al. primarily initiated treatment on the right. Fatigue improvement was measured using various self-reported questionnaires, including the FSS, PROMIS-29, and SF-36. All of these assessments relied on subjective patient data and lacked control groups. Despite these differences, there was still an overall 69.8% improvement reported in fatigue across the studies included in this review.

### Post-Exertional Malaise

Three studies examined post-exertional malaise from long COVID [10, 11, 13]. Duricka et al. [10] noted that 7 of 7 patients reported improvement in post-exertional malaise after bilateral SGBs. Chiang et al. only noted 11 of 25 patients reported improvement but the intervention was unilateral SGB with a contralateral SGB offered 1–2 weeks later. It is unclear how many patients received more than one SGB, which may explain the difference when compared to Duricka et al. [10]. Between Duricka et al. [10] and Chiang et al., 56% of patients reported improvement in post-exertional malaise. Duricka et al. [11] noted a significant improvement in all nine individuals with post-exertional malaise in the overall cohort using the DSQ2.

### Sleep

Across five different studies examining sleep changes/disturbances from long COVID after SGB, results were mixed [10, 11, 13, 14, 16]. Wang et al., Pearson et al., and Duricka et al. [11] reported substantial subjective improvements in sleep problems, while Chiang et al. and Duricka et al. [10] observed minimal or no benefit, respectively, with Duricka et al. [10] actually studying different objective aspects of sleep metrics, including wakefulness after sleep onset, total sleep time, percent REM sleep, sleep fragmentation, and sleep apnea. Regardless, no measurable changes were found after weekly bilateral SGBs for three weeks. Most patients across these studies received two or more SGBs, suggesting that neither laterality nor number of injections consistently influenced sleep outcomes. Despite the variability in assessment tools and methodologies, there was still an overall 72.2% improvement in sleep changes/disturbances after SGBs.

## Symptom Cluster 4: Neurological

### Changes in Smell/Taste

Though four studies examined the effect of SGB on changes in smell/taste associated with long COVID, each had a different protocol for its intervention [10, 13, 14, 17]. Pearson et al. used a single SGB or bilateral SGB, while Duricka et al. [10] and Chiang et al. used bilateral SGB with varying lengths of time between them. Sowerby et al., notably, acquired their data from online forums and it is unclear how many SGBs patients received. All studies relied on patients’ subjective reports of improvement in smell/taste and did not utilize objective measures. Moreover, some studies focused strictly on parosmia while others generalized more broadly to any changes in smell and/or taste. Nevertheless, 56% reported some degree of benefit from SGB for changes in smell/taste associated with long COVID.

### Autonomic Dysfunction

As autonomic dysfunction is known to be one of the predominant symptoms in long COVID, four of the studies in this review tracked this metric [11, 14–16]. Wang et al., Levey et al., Duricka et al. [11], and Pearson et al. all found that their patients had improvement in various symptoms typically thought to be autonomic in nature. The symptoms associated with autonomic dysfunction were heterogeneous between the studies. It is notable that Levey et al. and Pearson et al. found that patients experiencing palpitations or tachycardia reported improvement, but there were different SGB protocols and follow-up times in these studies. This may suggest that laterality or timing may not be a huge factor if determining whether SGB would be helpful for a patient with tachycardia from long COVID. Between all studies, 77% of patients reported improvement in autonomic dysfunction symptoms, and Levey et al. demonstrated an objective improvement in heart rate, with a significant decrease 10 beats per minute after a single SGB.

### Dizziness

Dizziness was evaluated by three trials with mixed results [10, 13, 14]. Overall, 70% of patients reported improvement across the aggregated studies at varying degrees of follow up. All reports of dizziness and subsequent resolution/persistence were subjectively reported across all studies with no formal assessment or validation of improvement.

## Symptom Cluster 5: Respiratory

### Cough/Dyspnea

When looking at respiratory symptoms such as dyspnea and cough, Pearson et al. was the only study in this review to track improvement in these symptoms after SGB [14]. 80% of patients reported improvement in cough and 88% of patients reported improvement in dyspnea, but the protocol for this study was not consistent, as different patients received either unilateral or bilateral blocks. More studies will be needed to determine if laterality or multiple blocks matter in the reduction of respiratory symptoms.

## Symptom Cluster 6: Miscellaneous

### Cortisol Levels

Duricka et al. [11] was the only study to evaluate SGB effect on cortisol levels. Following staged bilateral SGB, there was no change in morning salivary cortisol at 2 weeks or 2 months after intervention, suggesting SGB may not significantly affect systemic cortisol levels in the context of long COVID.

### GI Symptoms/Diarrhea

Two studies examined GI-related symptoms from long COVID [13, 14]. Pearson et al. used a single SGB or bilateral SGB and focused solely on diarrhea, with 7 of 8 patients reporting resolution of diarrhea. Chiang et al. offered a unilateral SGB with a contralateral SGB offered 1–2 weeks later and focused on GI symptoms more broadly. Interestingly, only 2 of 6 patients reported improvement in GI symptoms, though it is again unknown if the patients with these symptoms received multiple SGBs or which specific GI symptoms they had. Between both studies, 64% reported improvement of GI symptoms.

### Subjective Relief

Two studies examined patients’ subjective relief of overall symptoms from long COVID [15, 16]. Levey et al. noted that 7 of 8 patients seen at follow-up reported subjective improvement for at least 6 weeks after a single SGB. Wang et al. noted that 15 of 17 patients reported subjective symptoms improvement at 4 weeks after bilateral SGB with one week between injections. This metric is somewhat redundant in this review as many of the symptoms covered were ascertained from patients’ subjective reports and not by objective measures. Nevertheless, these two studies explicitly reported patients’ sense of subjective relief and therefore they are noted here. Between both studies, 88% reported subjective improvement in symptoms related to long COVID.

### Other Miscellaneous Symptoms

Pearson et al. also tracked nonspecific symptoms including rash, fever, and menstrual cycle changes [14]. Most patients (> 80%) with these symptoms reported resolution after SGB, though the sample sizes were extremely small (5 or less for each symptom).

#### Limitations

While this review demonstrates promising evidence that SGB is an effective treatment in alleviating some symptoms of long COVID, several limitations must be acknowledged. None of the included studies incorporated control groups, making it difficult to determine whether the improvement in patients’ symptoms reflected the treatment effect or natural recovery over time. Small sample sizes across these studies also further limit the generalizability of the results. Additionally, sampling and selection bias may have occurred, as some of the patients recruited were from the same clinics or similar patient populations. Publication bias may also play a role in this review, as most of the studies analyzed had a generally optimistic take on the procedure in the results.

Study designs and treatment protocols also varied substantially. Some studies used a single unilateral SGB, while others performed either sequential or bilateral blocks. In addition, some studies used fluoroscopic guidance, and others ultrasound guided. The injectate composition differed as well, as some studies used lidocaine alone while others combined lidocaine with dexamethasone.

**Fig. 1:**
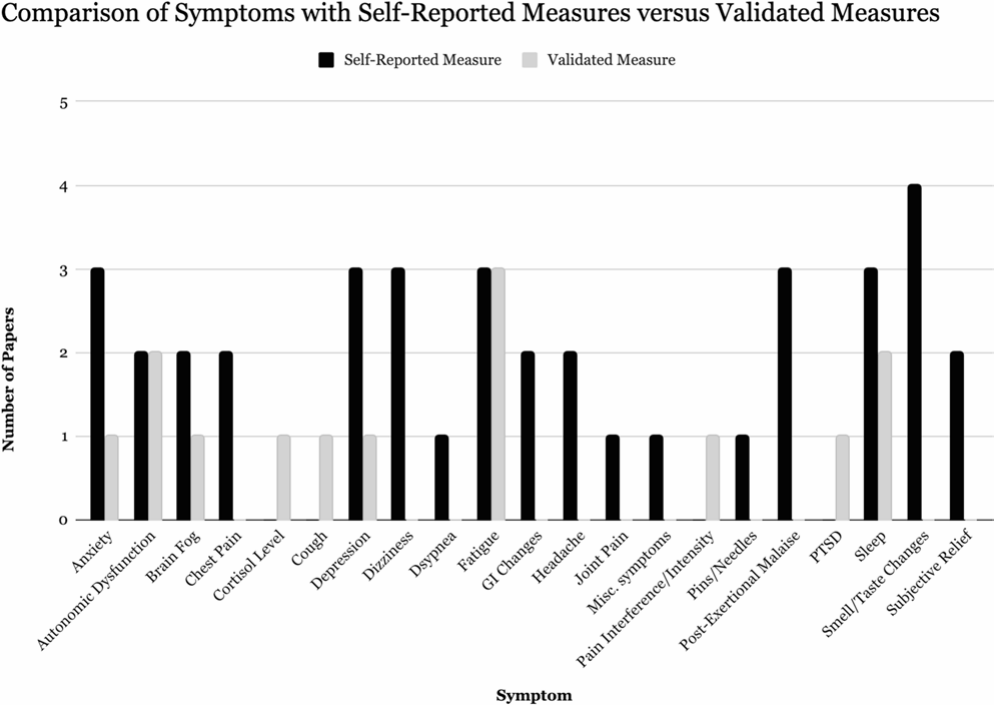
A bar graph comparing the number of papers evaluating each symptom by patient self-reporting or by validated measures

Another limitation noted was that most outcome measures were not validated or standardized and relied predominantly on self-reported surveys/questionnaires (Fig. 1). Figure 1 demonstrates the paucity of studies using validated measures to assess symptom improvement; with few exceptions, most symptoms were predominantly assessed via patient self reporting instead, introducing both recall and reporting bias. Follow-up duration and assessment intervals were not standardized either. Furthermore, long-term outcomes beyond 12 months were largely unexamined.

Altogether, these methodological limitations constrain the strength of the available evidence and underscore the need for larger, randomized-controlled trials to establish the efficacy and durability of SGB in managing long COVID symptoms. This paucity of evidence seems to be a theme within the long COVID research space, as existing literature on long COVID itself and the diagnostic criteria for the disease is limited [18].

Future studies on this modality should, at baseline, more clearly outline the outcome measures of the studies and do so in a randomized, controlled fashion with placebo control groups. These measures should include a more extended follow up period of somewhere between 6 and 12 months to determine the durability and longevity of treatment. Other trials should stratify the outcomes by symptom profile or disease severity, in order to better delineate which patients should receive this intervention. For further external generalizability, studies should determine if and which technique, laterality, or number of procedures leads to better outcomes. Lastly, as more research is performed on this disease and diagnostics advance, future studies should incorporate any imaging, biomarkers, or testing into outcome measures to assess response to SGB for their condition.

## Conclusion

Stellate ganglion block has emerged as a promising intervention for managing persistent symptoms associated with long COVID. Although the available literature remains limited, current evidence demonstrates consistent short-term improvements across multiple symptom domains, including fatigue, cognitive dysfunction, sleep disruption, and pain. As symptomatic management and therapy-based interventions remain the current standard of care, there is a pressing need for effective long COVID treatments, notably for those with refractory symptoms. In such cases, clinicians should consider SGB as an adjunctive option, particularly for individuals with prominent autonomic dysfunction - such as tachycardia, dizziness, dyspnea, gastrointestinal changes, and brain fog. Larger, controlled, and longitudinal studies are needed to establish the long-term efficacy, safety, and durability of SGB as a therapeutic strategy for long COVID. While preliminary evidence suggests potential benefit, definitive evidence will require identification of optimal treatment protocols and clarification of which patient subpopulations would derive the greatest benefit from this procedure.

## Data Availability

All data supporting the findings of this study are available within the paper, particularly in Table 1. Table 1 identifies all symptoms evaluated after stellate ganglion block by the various included papers, with their corresponding treatments and reported outcomes.

## Declarations

### Declarations

The authors have no corporate sponsorships to disclose.

## References

[1] Burrowes SAB, Casey SM, Pierre-Joseph N, et al. COVID-19 pandemic impacts on mental health, burnout, and longevity in the workplace among healthcare workers: a mixed methods study. J Interprof Educ Pract. 2023;32:100661. 10.1016/j.xjep.2023.100661. 37305404 10.1016/j.xjep.2023.100661 PMC10248469

[2] Malik P, Patel K, Pinto C, et al. Post-acute COVID-19 syndrome (PCS) and health-related quality of life (HRQoL)-a systematic review and meta-analysis. J Med Virol. 2022;94(1):253–62. 10.1002/jmv.27309. 34463956 10.1002/jmv.27309 PMC8662132

[3] Thant TM, Khandai AC, Gillan A, Peace M, Quinn D, Levenson J. Neuropsychiatric symptoms of subacute and chronic long covid. Am J Psychiatry. 2025;182(5):498–9. 10.1176/appi.ajp.25182003. 40308109 10.1176/appi.ajp.25182003

[4] Post covid-19 condition (long covid). World Health Organization. Accessed December 3. 2025. https://www.who.int/europe/news-room/fact-sheets/item/post-covid-19-condition

[5] Crook H, Raza S, Nowell J, Young M, Edison P. Long covid—mechanisms, risk factors, and management. BMJ. 2021;374:n1648. 10.1136/bmj.n1648. 34312178 10.1136/bmj.n1648

[6] Cheng AL, Herman E, Abramoff B, et al. Multidisciplinary collaborative guidance on the assessment and treatment of patients with long COVID: a compendium statement. PM&R. 2025;17(6):684–708. 10.1002/pmrj.13397. 40261198 10.1002/pmrj.13397 PMC12162235

[7] Researching COVID. to enhance recovery. RECOVER. Accessed December 3, 2025. https://recovercovid.org/about

[8] Feigin G, Velasco Figueroa S, Englesakis MF, D’Souza R, Hoydonckx Y, Bhatia A. Stellate ganglion block for non-pain indications: a scoping review. Pain Med. 2023;24(7):775–81. 10.1093/pm/pnad011. 36727500 10.1093/pm/pnad011

[9] Lipov EG, Joshi JR, Sanders S, Slavin KV. A unifying theory linking the prolonged efficacy of the stellate ganglion block for the treatment of chronic regional pain syndrome (CRPS), hot flashes, and posttraumatic stress disorder (PTSD). Med Hypotheses. 2009;72(6):657–61. 10.1016/j.mehy.2009.01.009. 19237252 10.1016/j.mehy.2009.01.009

[10] Duricka D, Liu L. Reduction of long COVID symptoms after stellate ganglion block: a retrospective chart review study. Auton Neurosci. 2024;254:103195. 10.1016/j.autneu.2024.103195. 38901177 10.1016/j.autneu.2024.103195

[11] Duricka DL, Liu LD. Stellate ganglion block reduces symptoms of SARS-CoV-2-induced ME/CFS: a prospective cohort pilot study. Fatigue Biomed Health Behav. 2025;13(2):97–114.

[12] Altmann DM, Whettlock EM, Liu S, et al. The immunology of long COVID. Nat Rev Immunol. 2023;23:618–34. 10.1038/s41577-023-00904-7. 37433988 10.1038/s41577-023-00904-7

[13] Chiang MC, Satko KM, Shin C, et al. Stellate ganglion block for the management of long COVID symptoms: a retrospective cohort study. Cureus. 2025;17(7):e88680. 10.7759/cureus.88680. 40861640 10.7759/cureus.88680 PMC12374758

[14] Pearson L, Maina A, Compratt T, Harden S, Aaroe A, Copas W, et al. Stellate ganglion block relieves long COVID-19 symptoms in 86% of patients: a retrospective cohort study. Cureus. 2023;15(9):e45161. 10.7759/cureus.45161. 37711269 10.7759/cureus.45161 PMC10498998

[15] Levey AO, Chen GH, Ngyuen A, et al. The Effectiveness and Safety of Stellate Ganglion Block in the Treatment of Symptoms from Long COVID-19: A Pilot Study. Psychopharmacol Bull. 2024;54(4):8–17. PMID: 39263197; PMCID: PMC11385263. 39263197 10.64719/pb.4500 PMC11385263

[16] Wang S, Salway RJ, Nicklay M, Kuo J. Effectiveness of dual sympathetic blocks for sympathetically mediated symptoms in post-acute sequelae of SARS-CoV-2 (PASC): an open-label, non-randomized pilot study. Cureus. 2025;17(3):e81530. 10.7759/cureus.81530. 40308396 10.7759/cureus.81530 PMC12042720

[17] Sowerby LJ, Almubarak Z, Biadsee A, Rocha T, Hopkins C. Coronavirus disease 2019 related parosmia: an exploratory survey of demographics and treatment strategies. J Laryngol Otol. 2023;137(11):1256–60. 10.1017/S0022215123000713. 37194063 10.1017/S0022215123000713 PMC10627779

[18] Seo JW, Kim SE, Kim Y, et al. Updated Clinical Practice Guidelines for the Diagnosis and Management of Long COVID. Infect Chemother. 2024;56(1):122–57. 10.3947/ic.2024.0024. 38527781 10.3947/ic.2024.0024 PMC10990882

